# The Pathological and Surgical Treatment of Suppurative Tonsilitis

**Published:** 1891-03-28

**Authors:** 


### SURGERY.

### THE PATHOLOGY AND SURGICAL TREATMENT OF SUPPURATIVE TONSILLITIS.

From the position of the tonsils it is obvious that very little material, either solid or liquid, can pass down the throat without coming into close contact with them, and this is, of course, still more the case if they are enlarged. If, therefore, they have active powers of absorption, their influence may be very considerable, either for good or ill. A microscopic examination of these organs shows a slight fibrous stroma, a large quantity of lymphoid tissue with aggregated masses of cells closely resembling Peyer's patches in the intestine, a copious supply of blood-vessels and lymphatics, and a lining of laminated, flat epithelium on the surface. There is, therefore, every requisite for rapid absorption, with the one exception of the epithelial covering. This, however, dips down into deep "crypts," and these recesses have, frequently, a layer of cells on the surface which is too thin to offer much resistance to diffusion. Moreover, a large number of wandering leucocytes are generally seen near the surface, ready to carry in small particles which they may attack. These facts, combined with clinical observations, have led Dr. Clarence Rice (*Medical Record*, January 31st, 1891) to come to the following conclusions: (1) That all kinds of tonsillar inflammation are due to septic causes; (2) that normally the leucocytes above-mentioned resist the entrance of pathogenic germs, but that when the organ is abnormal, entrance is easy; (3) that these affections may be all classified as either (*a*) follicular, (*b*) parenchymatous, or (*c*) peritonsillar; (4) that suppuration occurs in the connective tissue round these organs, and rarely in the tonsils themselves; and (5) that when the tonsils are liable to attacks of inflammation, they should be destroyed by the galvano-cautery or by other measures.

With these dicta most physicians and surgeons would probably be inclined to agree; the power of septic absorption possessed by these bodies is only too obvious; diphtheria and the various forms of tonsillitis show this; and when the delicate layer of protective epithelium is destroyed or stretched this must be doubly easy. It may be that the high temperature and prostration of ordinary quinsey are due to this cause, for the symptoms far exceed the apparent local inflammation and suppuration.

The "surgical treatment" of these affections is evidently one of prevention rather than cure; if the organ liable to disease is removed there is an end of the trouble in the future. But simple as the removal of part of the tonsils by the ordinary guillotine, by scissors and forceps, or by galvano-cautery would appear to be, it has never been practised to a sufficient extent. Objections are raised by the patient which are not decidedly opposed by the medical attendant; and for years the voice and general health are allowed to suffer from recurrent attacks of tonsillitis and the secondary involvement of neighbouring parts. It is well, therefore, that from time to time the importance of operative interference should be strenuously advocated.

Of the methods at our disposal Dr. Rice seems to advocate the galvano-cautery. This possesses the one advantage of preventing hæmorrhage, but it is slightly more formidable to the patient, more painful and slower than the method of removal by the guillotine. Of the enormous number of such operations carried out each year in hospitals there are very few records of serious hæmorrhage, for it is only necessary to remove a comparatively small portion of the diseased tonsil. The pain is trifling, and can be considerably lessened by the application of a cocaine spray; the cutting instrument should be sharp and the operation rapid. Too much is often taken away, and in cases of fibrous thickening of the gland, where the vessels are apt to gape, considerable hæmorrhage may follow from too free removal. That great clumsiness has occurred is evident from the fact that Velpean has recorded no less than four cases of wound of the internal carotid artery. Under ordinary precautions there is little or no danger. The improvement in health which follows this simple operation is very striking.

